# Sub-lethal signals in the mitochondrial apoptosis apparatus: pernicious by-product or physiological event?

**DOI:** 10.1038/s41418-022-01058-0

**Published:** 2022-09-21

**Authors:** Georg Häcker, Aladin Haimovici

**Affiliations:** 1 Institute of Medical Microbiology and Hygiene, Medical Center, University of Freiburg, Faculty of Medicine, Freiburg, Germany; 2 BIOSS Centre for Biological Signalling Studies, University of Freiburg, Freiburg, Germany

**Keywords:** Inflammation, Cell biology, Cell death and immune response

## Abstract

One of the tasks of mitochondria is the rule over life and death: when the outer membrane is permeabilized, the release of intermembrane space proteins causes cell death by apoptosis. For a long time, this mitochondrial outer membrane permeabilization (MOMP) has been accepted as the famous step from which no cell returns. Recent results have however shown that this quite plainly does not have to be the case. A cell can also undergo only a little MOMP, and it can efficiently repair damage it has incurred in the process. There is no doubt now that such low-scale permeabilization occurs. A major unclarified issue is the biological relevance. Is small-scale mitochondrial permeabilization an accident, a leakiness of the apoptosis apparatus, perhaps during restructuring of the mitochondrial network? Is it attempted suicide, where cell death by apoptosis is the real goal but the stimulus failed to reach the threshold? Or, more boldly, is there a true biological meaning behind the event of the release of low amounts of mitochondrial components? We will here explore this last possibility, which we believe is on one hand appealing, on the other hand plausible and supported by some evidence. Recent data are consistent with the view that sub-lethal signals in the mitochondrial apoptosis pathway can drive inflammation, the first step of an immune reaction. The apoptosis apparatus is almost notoriously easy to trigger. Sub-lethal signals may be even easier to set off. We suggest that the apoptosis apparatus is used in this way to sound the call when the first human cell is infected by a pathogen.

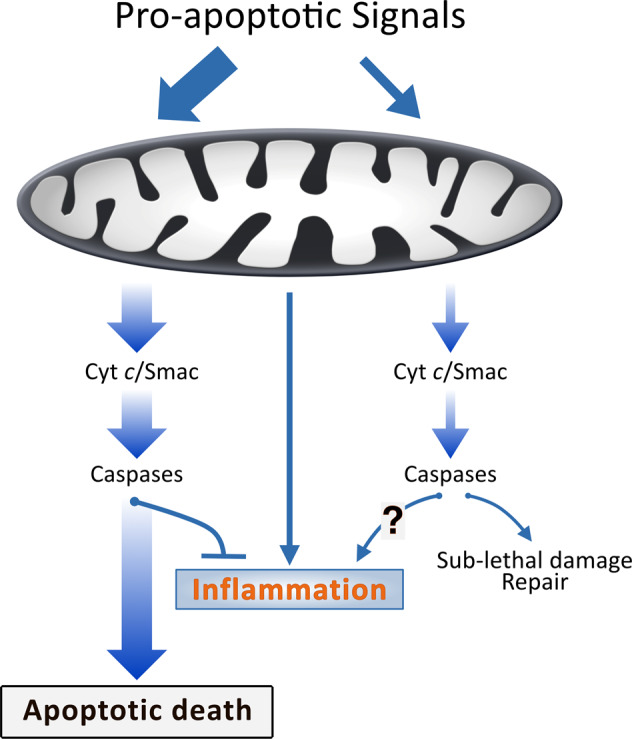

## Facts

- During apoptosis, generalized permeabilization of the mitochondrial outer membrane (MOMP) releases caspase-activating proteins. At later stages, the inner membrane ruptures and can release mitochondrial DNA (mtDNA).
- There is the possibility that MOMP stays incomplete, causing the activation of sub-lethal levels of caspases. The cell can recover but may incur genomic mutations and show signs of aggressive growth.
- Sub-lethal signals have been detected with a number of experimental and chemotherapeutic stimuli but also during microbial infection. Previously described instances of low-level caspase-activity in non-apoptotic cells are probably also generated by such signals.

## Open questions

- What is, if any, the physiological relevance of sub-lethal signalling in the apoptosis pathway?
- Recent work suggests that these signals can contribute to inflammation, suggesting the release of mitochondrial factors. Which factors drive inflammation?
- Does sub-lethal signalling in the mitochondrial pathway involve the complete permeabilization of a small share of the mitochondrial network or the release of a small share of mitochondrial components by perhaps a larger part of the network?

## Introduction

Mitochondrial apoptosis occurs when the mitochondrial outer membrane is permabilized (the term MOMP, for mitochondrial outer membrane permeabilization is commonly used), and intermembrane space proteins are released into the cytosol, most importantly cytochrome *c* and Smac. During apoptosis, the release of these proteins is swift and extensive or even complete [1–3]. Caspases are activated and the cell dies. Actual cell death—measured most easily by the inability to resume growth—is perhaps most closely coupled to mitochondrial function [4, 5] and correlates with the loss of mitochondrial membrane potential [6]. Caspases cause much of the morphological changes of apoptosis and are involved in the disposal of the dead cell, but a caspase-function that may be more important in the intact organism than actual cell death is the inhibition of inflammatory activity in the dying cell [7]. Mitochondria harbour inflammatory potential: if mitochondria are permeabilized by Bax/Bak and caspases are inhibited or deleted, the cell produces type I interferon [8, 9] and NF-κB-dependent cytokines [10], and apoptosis becomes inflammatory (although, somewhat paradoxically, Apaf-1-deficient mice on some genetic backgrounds survive without apparent abnormality [11]). While this is cell biologically very interesting, it raises a fundamental question: in which situation does such inflammatory cell death occur? There are a few reports where caspase-activation in cancer cells may be altered, mostly by down-regulation of Apaf-1 in melanoma (although with unknown frequency [12]) and in pancreatic cancer [13], and cardiomyocytes have been reported to express little Apaf-1 and to generate only low caspase-activity [14]. However, a lack of caspases or a block of caspase-activation downstream of mitochondria seems very rare in human cells, and the non-inflammatory nature of apoptotic cell death has for decades been regarded as a hallmark of apoptosis [15]. In other words, inflammation through the release of mitochondrial components occurs at least only very infrequently outside experimental caspase-blockade. Indeed, it is so rare that it was not discovered until 2014. Why then do we have such a system in our cells that is apparently dedicated to triggering inflammation yet is normally inactive?

Enter the concept of sub-lethal signalling in the apoptosis apparatus. In retrospect, there have been numerous earlier reports that apoptosis-signalling can occur in cells that are not dying. The majority of the published reports on this subject have described caspase-activity in non-apoptotic cells. The recognition of such signals by the wider community, and their detailed cell biological analysis however is a more recent and still ongoing development. Small-scale, sub-lethal signalling of the apoptosis apparatus has been described originating from the TRAIL death receptor [16] and from signals of the mitochondrial apoptosis pathway [17]. During mitochondrial apoptosis, the whole network of mitochondria undergoes MOMP, but there is the possibility of only a few individual mitochondria, which appear to have fissioned off the network, to permeabilize and to release cytochrome *c* [17, 18]. The term ‘minority MOMP’ has been coined to describe this event [17]. The amount of cytochrome *c* released in this situation is too small to activate lethal amounts of caspases, and the share of the mitochondrial network damaged is too small to endanger survival of the cell. The downstream event most sensitively detected is a DNA-damage response, a consequence of DNA-damage through the caspase-activated DNAse (CAD) [17, 19]. CAD is normally kept inactive by the inhibitor ICAD until ICAD is inactivated by caspases [20]. CAD can cause permanent genomic mutations [16, 17] with the obvious associated risks but otherwise a cell has a great capacity of recovering from transient, sub-lethal signals [21, 22].

**Fig. 1:**
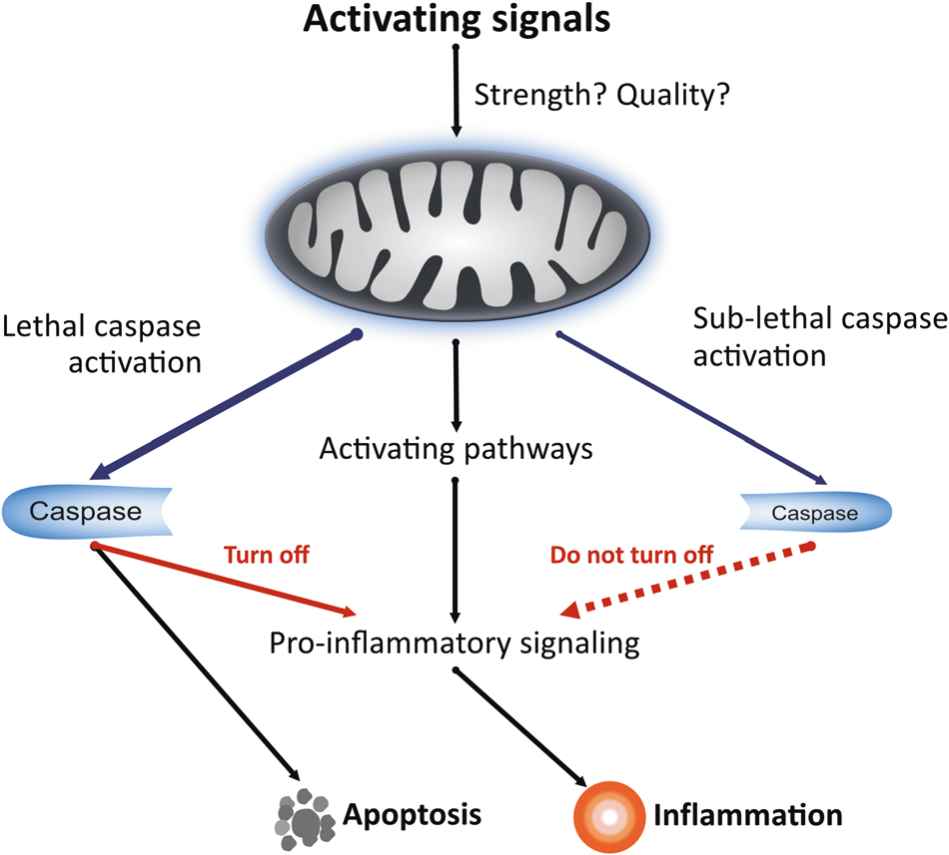
Model of activating and inactivating pathways in apoptosis and sub-lethal mitochondrial signalling. The signals upstream of mitochondria are often molecularly uncertain; signals that induce apoptosis vs. sub-lethal signalling may simply differ in strength or have different qualities. Stimulating signals are generated but during apoptosis caspase-activity turns them off, leading to non-inflammatory apoptotic cell death. If the caspase-activity is low, such as during minority MOMP, the inflammatory activity will persist, and the cell will indicate the signalling to the organism by the production of inflammatory mediators.

This is all fascinating but is there a point to it? All the reported consequences of sub-lethal signalling in the apoptotic pathway seem deleterious and undesirable. Sub-lethal signalling can cause the accumulation of mutations in the genome [16, 17], and cancer cells that have experienced these signals, or where the pathway is spontaneously active, show an increase in growth and aggressiveness [22–25]. We will argue that this mechanism of small scale mitochondrial signalling is the answer to the question why cells have the pro-inflammatory machinery downstream of mitochondria. In this model, mitochondria can through such small-scale activation of the apoptosis apparatus signal in various ways and in fact contribute to life as a complex organism. One function of these signals seems to be cellular differentiation (see below). We propose that a second function is immune alert and the recognition of danger. As discussed above, mitochondria can release pro-inflammatory signals and stimuli. Crucially, the low level of caspase-activity induced by the sub-lethal signals is, in this hypothetical model and supported by some experimental evidence [19, 26], too low to turn off the activating signals generated by mitochondria (Fig. 1). In this way, the well-known promiscuous response of mitochondria towards many stimuli may translate into a comprehensive ability to sense danger and injury.

## Evidence of sub-lethal signals in the apoptosis apparatus

Even though sub-lethal signals in the apoptosis apparatus are receiving increasing attention, evidence of the existence of such signals dates back more than two decades. Most earlier reports of what we now may perceive as sub-lethal signalling in the apoptosis pathway (‘sub-lethal signalling’ for short) concern caspase-activity, or activation of a specific cell death-caspase in the absence of cell death (reviewed in [27–29]). CAD-activity in differentiating myoblasts [30] and in cells undergoing prolonged mitotic arrest [31] has also been described in sub-lethal settings. In most of these cases, the upstream signals have not been worked out but given recent data it seems a fair guess that partial mitochondrial permeabilization is involved.

The reported examples of caspase-activity in the absence of cell death mostly describe situations of cellular differentiation. There are numerous studies reporting non-apoptotic roles of caspases that are known for their role in apoptosis, in mammals mostly caspase-3, and often in organismal development and cellular differentiation (reviewed in [28]). Non-lethal caspase-activity has perhaps been most comprehensively studied in *Drosophila* development. Regulation of caspase-activation and of apoptosis is substantially different between flies and mammals. Most strikingly, in *Drosophila* the caspase-activating signal is not mitochondrial permeabilization but antagonism of the caspase-inhibitory IAPs by dedicated proteins [32]. However, despite these differences sub-lethal caspase-activation can also occur in *Drosophila*. A number of reports have identified non-lethal caspase-activity in sperm-cell generation [33, 34] and in the Wingless differentiation pathway [35, 36]. Recently, a cellular reporter of past caspase-activity was expressed in *Drosophila*. This reporter identified widespread caspase-activation in developing cells that lived to adulthood [37], indicating that numerous cells contained caspase-activity at some stage during their development but lived on to form adult tissues.

A substantial number of reports have further shown non-apoptotic activation of caspases in mammalian tissues [7]. Caspases of the apoptosis pathway are required for mammalian neuronal development [38] and in tuning neuron-function, for instance by axon-pruning [39, 40]. Non-lethal effector caspase-activity appears to be a feature of the differentiation of many types of mammalian stem cells: embryonic stem cells [41], iPSC [42], bone marrow stromal stem cells [43] and neuronal stem cells [44] all appear to depend for their differentiation so some extent on caspases; in haematopoietic stem cells, caspase-3 contributes to quiescence [45]. For the differentiation of muscle cells from myoblasts, caspase-9 [46], caspase-3 [47] and CAD [30] are all required, suggesting a non-apoptotic signalling pathway downstream of mitochondria. No defect in muscle-development has however been reported for the gene-deficient mice, suggesting that other signals can compensate for the defect in vivo. Roles of sub-lethally active caspases in tumour development have also recently been reviewed [48]. It has mostly not been established what the upstream signals are that activate caspases in these situations, and it is often molecularly unclear how caspase-activity drives the biological responses reported. It seems however almost inescapable to conclude that this caspase-activation is a result of sub-lethal signalling in the apoptosis pathway. We have recently reported evidence of continuous sub-lethal activity of the mitochondrial apoptosis pathway in human cell lines in culture, where these signals drove growth behaviour of the cells [24]. Table 1 summarizes these biological functions. These examples make it likely that the mitochondrial apoptosis apparatus is prone to signal in the absence of cell death, and one function may be in development and differentiation.

**Table 1:** Evidence for sub-lethal signaling in eukaryotic cells.

Biological situation/function	Signaling component	References
Drosophila		
Sperm development	Caspases	[25, 26]
Sensory organ precursor development	Caspases	[27, 28]
Mammals		
Neuronal development	Caspases	[30–32]
Stem cell differentiation	Caspases	[33–36]
Muscle cell differentiation	Caspases, CAD	[23, 37, 38]
Cancer and metastasis	Caspases, CAD	[12, 13, 17, 19, 20]
Inflammation	mtDNA, Smac	[15, 47]
Reviews covering these events		[22, 61]

## A possible function of mitochondrial sub-lethal signals in immune alert

**Fig. 2:**
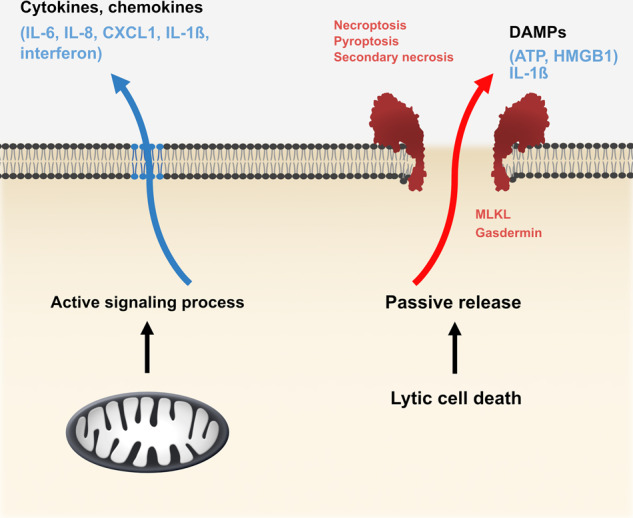
Two ways to induce inflammation by cell death-signalling. ‘Lytic’ cell death, such as necroptosis and pyroptosis, cause plasma membrane rupture and the passive release of cellular inflammatory molecules (damage-associated molecular patterns, DAMPs) such as ATP or high mobility group protein B1 (HMGB1). Sub-lethal mitochondrial signals can induce the secretion of specific mediators of inflammation, in particular cytokines and chemokines that will attract and activate immune cells, in the absence of cell lysis. During pyroptosis both processes occur: the active secretion of IL-1β and the passive release of cellular factors.

Inflammation is the first stage of the immune response. Inflammation is a complex affair, with numerous players and a wide array of mediators. That apoptosis is non-inflammatory has been the widely held view even before much was known about which stimuli drive inflammation in the context of cell death [49]. For the purpose of categorizing inflammation during cell death signalling, we find the division of the inflammatory stimuli into two classes helpful. One class are stimuli that are released by the dying cell upon plasma cell rupture, in particular during other cell death forms such as pyroptosis and necroptosis (commonly referred to as damage-associated molecular patterns, DAMPs [50]). The other class are cytokines and chemokines that are secreted by the signalling cell (Fig. 2). These messenger molecules can have strong inflammatory effects, for instance by recruiting neutrophils or by increasing capillary permeability. During sub-lethal signalling, there is no plasma cell rupture and probably no release of DAMPs, but there may be the secretion of cytokines (and chemokines). If a cell infected by a microbial pathogen secretes for instance the chemokine IL-8, neutrophils will be attracted to the infected cell, and IL-8 is one factor to whose secretion sub-lethal signalling can contribute [19].

We will first have to consider the relationship of infectious agents and apoptosis; is apoptosis a feature of infection, and may therefore sub-lethal signalling also occur? There are many instances where the infection by microbial agents has been described to affect apoptotic processes in some way. The first description of a virus blocking apoptosis (in insect cells) dates back over 30 years [51]. Many viruses carry genes whose products inhibit apoptosis, and the ability of viruses to control mitochondrial apoptosis is very well documented [52]. This need of viruses to inhibit apoptosis implies that there is apoptosis to be inhibited during infection. In the most straightforward scenario, this means that the virus at the same time induces apoptosis, or, looking at it from the cell’s perspective, the cell senses viral presence and tries to undergo apoptosis. This has in fact been shown directly in a number of viral infections (see for instance [51, 53]). Infection with at least some viruses thus appears to have pro-apoptotic effects.

Perhaps the situation is more complicated for bacteria, because the signalling between bacteria and vertebrate cells tends to be more complex and less well understood. Although apoptosis can be induced by bacteria in cell culture, especially when using high bacterial numbers, it is difficult to know what that means in physiological circumstances. Bacteria are so heterogeneous that it makes no sense to speak of ‘the bacterium’, and the behaviour of different bacterial species regarding apoptosis may be very different. Nevertheless, in vitro-studies find at least weak pro-apoptotic activity for many of the bacteria that are frequent objects of research, such as *E. coli*, *Listeria*, *Helicobacter*, various species of *Streptococci* or gonococci; the list is long, and in a number of cases pro-apoptotic activity can be linked to bacterial products [54, 55]. On the other hand, bacteria colonize all accessible surfaces of the human body, and the detection of bacteria by vertebrate host cells does, at least initially, not make the distinction between colonizer and pathogen. Therefore, full-blown apoptosis cannot be the outcome of this interaction, but sub-lethal signals remain a possibility. Indeed, we have recently found in vitro that infection of epithelial cell lines and fibroblasts with not only any of the three viruses tested but also the two bacterial species, as well as one protozoan parasite, triggered sub-lethal signals at the mitochondria [19]. We believe that this is relevant, because the signals generated in the apoptotic pathway contributed to the secretion of soluble pro-inflammatory products [19, 56]. As we will discuss further below, such signals may be a convenient way for the organism to initiate an inflammatory and immune response.

**Fig. 3:**
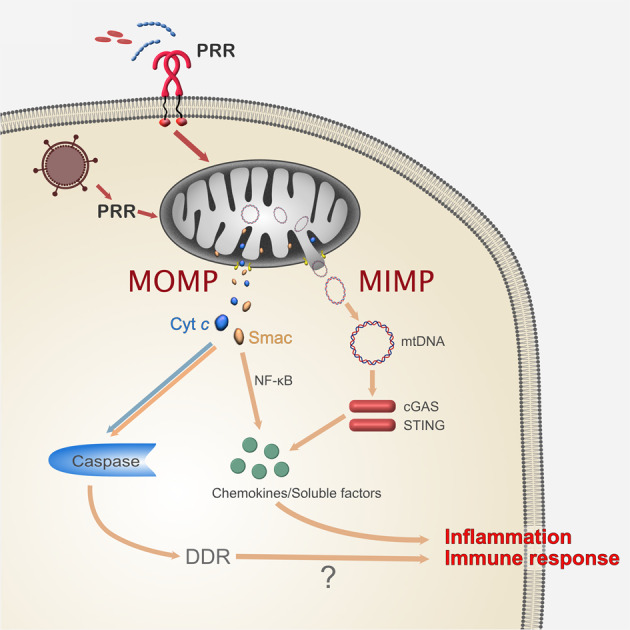
Pro-inflammatory signals through during sub-lethal mitochondrial activity. Viruses and bacteria are initially recognized by cellular pattern recognition receptors, which may recognize common bacterial molecules such as peptidoglycan or viral nucleic acids. PRR can have the capacity to activate mitochondrial apoptosis signals, leading to (complete or presumably also partial) mitochondrial outer membrane permeabilization (MOMP). Whether they can also induce mitochondrial inner membrane permeabilization (MIMP: herniation following MOMP) is uncertain. MOMP will release intermembrane space proteins, while MIMP is required for mtDNA-release. Smac can activate NF-κB and can contribute to the secretion of soluble factors; mtDNA can activate cGAS to produce the stimulating ligand for STING. As long as caspase-activity is low enough, it will not counteract the pro-inflammatory activity but may in fact through the activation of CAD cause a DNA-damage response with potentially inflammatory activity. mtDNA can be recognized by cGAS and activate STING.

How could this work? The microbe has to be detected by cellular receptors, and these receptors have to trigger sub-lethal signalling. It is notoriously difficult to link upstream signals to the Bcl-2-family during apoptosis, but there are some hints of a possible triggering mechanism in the literature. During infection of HeLa cells with *Shigella* bacteria, cleavage of the pro-apoptotic Bcl-2-family member Bid caused the release of Smac from mitochondria in the absence of cell death [57]. This is most unusual, as in other situations Smac and cytochrome *c* are released together [3]. The upstream receptor activating Bid is unclear but the mechanism involves calpain-dependent cleavage [57]. There is however an intriguing possibility of receptors triggering sub-lethal signalling, namely pattern recognition receptors (PRR) (Fig. 3). PRR are receptors that recognize classes of microbes through conserved molecules and that fall into a number of different classes [58]. PRR are well known for their function in alerting the cell to the presence of microbes: signals from PRR typically activate the main modules of innate immunity, NF-κB and type I interferon [59]. On top of this, there is another signalling quality that appears to unite PRR from various classes: a substantial number of PRR have been reported to have pro-apoptotic potential.

Descriptions of apoptosis triggered by PRR go back more than 20 years, when the principal cell-killing activity of the PRR Toll-like receptor (TLR) 2 was reported [60, 61]. Through which pathway TLR2, which signals through the adapter MyD88, induces apoptosis is still molecularly unclear. TLRs that use the adapter TRIF, TLR3 and −4, are better understood in this respect because TRIF can utilize the TNFR1-signal transduction machinery and therefore induce apoptosis in a similar fashion to TNF [62]. TLR3/4 can induce apoptosis in macrophages [63], while TLR3 is more widely expressed than TLR4 and can kill for instance melanoma cells [62]. Pro-apoptotic activity of another class of PRRs, RIG-like helicases RIG-I and Mda5 (cytosolic sensors of unusual entities of RNA), has also been reported [64]. When overexpressed, the cytosolic sensor of peptidoglycan, NOD1 – which recognizes a breakdown product of bacterial cell walls and can signal the presence of cytosolic bacteria –, can activate caspase-9 and kill the cell [65]. The cGAS/STING (cyclic GMP-AMP-synthase/Stimulator of Interferon Genes) module of microbial DNA-sensing can likewise induce apoptosis in some cells [66, 67]. It is unlikely that apoptosis-induction is a major function of PRR, and demonstrating this activity requires specific experimental conditions. However, it seems worth considering the possibility that sub-lethal rather than lethal signals originating from PRRs are part of the pro-inflammatory signalling network. Although there is still not much work that would directly have tested these ideas, literature does suggest that sub-lethal mitochondrial signalling occurs frequently in infection. If these signals have qualities that induce the secretion of cytokines/chemokines, then sub-lethal signalling may be inflammatory. In the next section, we will consider the signalling qualities that mitochondria may have and signalling mediators that may be released from mitochondria.

## Potentially stimulatory mitochondrial components

Mitochondria have almost certainly begun their life as endosymbionts when an archaeon engulfed a bacterium [68, 69]. Although mitochondria still share with gram-negative bacteria the building plan of two membranes and solute-flux across the outer membrane through β-barrel proteins, they do not contain the stimulatory lipids of the outer bacterial membrane (LPS) or the peptidoglycans of the bacterial cell wall, whose breakdown fragments can be recognized by NOD1/2. They do however still have nucleic acids that can stimulate PRR within the cell and can in principle release both RNA and DNA. Another possibility to stimulate the cell are intermembrane space proteins such as Smac/DIABLO, which may also activate cytosolic signalling. Lastly, it may also have to be considered that the DNA-damage response (DDR) that is caused by the activity of CAD during sub-lethal signalling may have pro-inflammatory effects. We will briefly look at the identified activity of these molecules before discussing their potential role in sub-lethal mitochondrial signalling (Fig. 3).

### mtDNA

mtDNA is the inflammatory mediator that has most frequently been put forward in the discussion of mitochondrial pro-inflammatory activity. Mitochondria have numerous copies of circular DNA. Cytosolic double-stranded DNA is recognized by the cyclic GMP-AMP-synthase (cGAS), which then produces a stimulating ligand for the stimulator of interferon genes (STING). STING-activity drives pro-inflammatory signalling and the production of both IFN and NF-κB-dependent factors [70]. cGAS and STING are expressed in most tissues [71], so it may operate as a recognition system in non-professional immune cells. Cytosolic mtDNA can also activate the inflammasome in cells expressing the components of this complex (reviewed in [72]). It is therefore a very appealing model that the release of mtDNA and its subsequent recognition by cGAS is an alert system registering sub-lethal mitochondrial signals. One point to be considered is the mechanics of mtDNA-release. mtDNA is located in the mitochondrial matrix, and during apoptosis the death-inducing event is the permeabilization of the outer membrane, which will leave the matrix untouched. At later stages, however, the inner membrane may also rupture, leading to the release of mtDNA [73, 74].

Release of mtDNA into the cytosol has been discovered upon stress caused by aberrant DNA-packaging [75] and during (full-blown) apoptosis [8, 9]. It is a late event in apoptosis that the mitochondrial inner membrane herniates as a consequence of the formation of Bax/Bak pores in the outer membrane, enabling the escape of mtDNA into the cytosol [73, 74] (late in the sense that it only occurs once MOMP has been completed). If autophagy is blocked and aged mitochondria are therefore not removed, mtDNA can also be released into the cytosol, presumably from such dysfunctional mitochondria [76]. These examples all suggest that individual mitochondria that are damaged either by the apoptosis apparatus or by ‘aging’ can release mtDNA into the cytosol. In principle, pathologically accumulating mitochondrial dsRNA can also escape into the cytosol and can activate inflammatory pathways in a Bax/Bak-dependent fashion [77] but if this has any bearing on apoptotic signalling is not clear. Finally, senescent cells secrete a number of cytokines and chemokines, a condition referred to as the senescence-associated phenotype (SASP). The SASP has been linked to mitochondria [78], and it seems worthwhile considering that the continuous release of mitochondrial components may contribute to this inflammatory condition (indeed, recent work implicates mtDNA in senescence-associated inflammation; Stephen Tait, personal communication).

### Smac and other intermembrane space proteins

Smac/DIABLO is a mitochondrial intermembrane space protein that was identified as an interaction partner and antagonist of inhibitor of apoptosis proteins (IAPs), a cytosolic class of ubiquitin ligases [79, 80]. More IAPs with possibly other undiscovered functions exist, but the ones that have been well characterised (and are known to be antagonized by Smac) are XIAP and cIAP1/2. XIAP has a non-apoptotic signalling role in NF-κB-induction through the NOD-pathway [81], as well as functions in controlling the activation of the apoptosome and caspase-9 during apoptosis [82, 83]. cIAP1/2 are particularly important in regulating signal transduction through TNFR1 and in the activation of alternative NF-κB [84, 85].

We do know the cytosolic activities of Smac, partly because of studies with the specific, experimental expression of cytosolic Smac, but mostly through the use of small-molecule Smac-mimetics [86]. If Smac is expressed experimentally in the cytosol (rather than being sequestered in mitochondria), it antagonizes cIAP1/2 and leads to the upregulation of the kinase NIK, followed by activation of alternative NF-κB and inflammatory target genes. Small molecule Smac-mimetics have similar effects [84, 87]. There is however no getting away from the fact that Smac itself in resting cells is at least mostly mitochondrial. It is released during apoptosis alongside cytochrome *c* [3] and is required at least in some cells for caspase-activation to a level sufficient for apoptosis [83]. But what about other potential functions, such as cIAP1/2-antagonism? It would appear at least doubtful that the Smac-dependent antagonism of cIAPs and the non-apoptotic functions of XIAP play a role upon apoptotic mitochondrial permeabilization when caspases are rapidly activated and the cell dies. If Smac was however released by sub-lethal mitochondrial activity, such signals would be conceivable. As discussed above, *Shigella*-infection caused the non-lethal release of Smac. During sub-lethal signalling, it is primarily the release of cytochrome *c* that has been investigated [17] but caspases are activated in this situation, and because apoptotic caspase-activity does often require Smac [83], it seems likely that caspase-activation during sub-lethal signalling also has an element of Smac-dependent XIAP-neutralization, and that Smac-release therefore also occurs. If Smac reaches the cytosol in these situations, a signalling function may therefore be expected. When apoptosis was induced and caspases were inhibited, NF-κB was activated. This could not be put down to Smac on its own [10], but there are more mitochondrial candidate proteins that may have the role of antagonizing cIAPs [88]. We have recently reported that during non-lethal infection of epithelial cells with the bacterium *Helicobacter pylori*, Smac was released even apparently in preference over cytochrome *c* and contributed to the activation of alternative NF-κB and to chemokine secretion from the cells [26]. This is clearly an area that requires more research, but Smac and Smac-like proteins are candidates for the function of pro-inflammatory molecules released by mitochondria during sub-lethal signalling in the apoptosis pathway.

### The DNA-damage response (DDR)

There is a further facet that has not been sufficiently explored. A regular occurrence during sub-lethal signalling in the apoptosis pathway appears to be the activation of CAD [16, 17, 19, 24, 25, 89]. The DDR that follows the activation of CAD in fact has been the most sensitive marker of sub-lethal signals to date [17, 19]. Other DNA-damaging stimuli induce inflammation: sunburn is at least to a substantial part the result of DNA-damage induced by UV-irradiation [90]. For chemically caused DNA-damage, parts of a pro-inflammatory signalling pathway have been worked out [91], and DNA-damage has a role in the immune response [92]. These pieces of evidence suggest that a DNA-damage that is inflicted by CAD during sub-lethal mitochondrial signals also has pro-inflammatory qualities. Indeed, in cancer cells the kinase ATM is active downstream of CAD and has been proposed to drive tumour growth [25], and possibly additional biological events [93]. Because the available data indicate that a CAD-dependent DDR is caused by microbial infection [19, 26], this could contribute to the initiation of an immune response to such pathogens. It may be a substantial conceptual step to accept that DNA-damage through sub-lethal signals is in fact desirable, despite its potentially deleterious consequences. On the other hand, DNA-damage through any endogenous or external agent is extremely common in daily life [94], and cells infected especially with viruses often don’t survive the infection and are no risk to the organism. It is therefore at least conceivable that the DDR, presumably almost invariably triggered by sub-lethal signals, is a component of inflammation.

## What is happening at mitochondria when the apoptosis apparatus is partially activated?

As discussed above, mitochondrial inflammatory activity has something ambivalent. Mitochondrial permeabilization can be pro-inflammatory, but this inflammatory activity is under the control of caspases, which again are activated by mitochondrial permeabilization [8–10]. Put more simply, MOMP is immunologically silent as it activates caspases to a very high level sufficient to turn off any inflammatory activity. There is evidence however that low-level mitochondrial permeabilization can cause the secretion of cytokines and chemokines [19, 26]. This suggests that a balance is possible where pro-inflammatory components are released but caspase-activity remains at a level too low to counteract inflammation.

Two principal ways may release small amounts of mitochondrial constituents. Mitochondria normally form a network that is constantly changing by fission and fusion: small parts of the tubular network bud off and form ‘individual’ mitochondria (fission), but portions of the network can also join by fusion. The first possibility of permeabilization is that individual mitochondria are completely permeabilized and empty their entire contents [18]. The second possibility is that larger parts of the network release limited pools of activating molecules. This difference is meaningful. Complete permeabilization may release all mitochondrial contents, including matrix molecules such as mtDNA and RNA [18]. Incomplete permeabilization is probably limited to the release of intermembrane space proteins such as cytochrome *c* and Smac (Fig. 3). It is of course also possible that both occur in different situations, or even in the same cell. This is however clearly a difference that matters, and it will be of relevance to make the distinction between these two possibilities in what may be physiological circumstances of sub-lethal signalling, especially in infection.

## Conclusion

It seems clear that sub-lethal signals can be generated in the apoptotic pathway, probably most commonly at mitochondria. Although caspases make apoptosis generally immunologically silent, the published examples strongly suggest that sub-lethal mitochondrial signals can provide an inflammatory signal. We are only at the beginning in the endeavour to understand this. It will be clear from this article that we are in favour of the possibility that sub-lethal signals do have a physiological function, most likely in the recognition of danger and injury, but probably also in cellular differentiation. Deficiency in major apoptosis signalling proteins can lead to embryonic death in mice, although some mice with defects in the main regulators of mitochondrial and death-receptor-linked apoptosis (as well as necroptosis) can survive [95] (recent data show that Bid can act as a mitochondrial effector [96], and Bid indeed can rescue some of the embryonic problems [97]. Perhaps there are more unidentified effector molecules). Although this is mere speculation, it is conceivable that the disturbance of sub-lethal signals can also contribute to this phenotype, as has already been proposed [7]. Negative consequences of sub-lethal signalling have been reported, in particular the introduction of genomic mutations. In chronic infections this may contribute to malignant transformation – which is a known feature of long-lasting or repetitive infection and inflammation [98] – but in the short term this appears unproblematic. If this proposed alert function is physiologically relevant, then it clearly has great potential. It is remarkable how easily cells can be triggered to undergo apoptosis. If, as seems the case, sub-lethal signals use the same pathways and components, sub-lethal signals in the apoptosis pathway may be even more easily generated and may be widespread indeed. In this way, the mitochondrial apoptosis apparatus would be put to use as a very sensitive trigger of inflammation and immune alert. Perhaps of particular importance could be the fact that all nucleated cells would be capable of using it. The immune response is perhaps more commonly thought of as the domain of specialised cells, such as myeloid cells and lymphocytes. The first contact with a pathogen that a human body has may however be the pharyngeal epithelial cell that is infected by a novel coronavirus. The ability of this cell to respond to this infection, by calling in the professionals through chemokine secretion, or possibly through an enhanced ability to restrict viral growth, may provide a necessary advantage in the race against viral replication and spread. Besides testing this hypothesis further, it will be interesting to study whether this is a function of mitochondria that has evolved along with their ability to release apoptogenic factors or whether such a mechanism is also found in organisms where apoptosis is initiated without mitochondrial permeabilization, for instance in insects.
